# Serological Survey of *Leptospira* spp. in Livestock and Rodents from Different Settlements in the Kilombero Wetland, Tanzania

**DOI:** 10.3390/pathogens13121059

**Published:** 2024-12-01

**Authors:** Mwajabu Selemani, Rhodes H. Makundi, Apia W. Massawe, Abdul S. Katakweba

**Affiliations:** 1 Institute of Pest Management, Sokoine University of Agriculture (SUA), Morogoro 67101, Tanzania; 2 Department of Wildlife Management, Sokoine University of Agriculture (SUA), Morogoro 67101, Tanzania; 3 The African Centre of Excellence for Innovative Rodents Pest Management and Biosensor Technology (ACE IRPM & BTD), Institute of Pest Management, Sokoine University of Agriculture (SUA), Morogoro 67101, Tanzania; rmakundi@yahoo.com

**Keywords:** leptospirosis, serovars, livestock, rodents, wetland, Tanzania

## Abstract

Background: The circulation of *Leptospira* has been linked to various occupational activities globally. This study investigated the seroprevalence of *Leptospira* spp. in rodents and livestock (cattle and goats) in three settlements/villages involved in agriculture, livestock keeping, and mixed agriculture and livestock in the Kilombero district, Tanzania. Methods: Data were collected during the wet and dry seasons. A total of 179 rodents were live-captured from selected habitats. Livestock samples were collected from 80 cattle in a livestock settlement and 120 goats from both livestock and mixed agricultural–livestock settlements. The microscopic agglutination test was utilized to identify *Leptospira* serovars. Results: The seroprevalence of *Leptospira* spp. was 17.3% in rodents (21.7% in *Mastomys natalensis* and 3.9% in *Rattus rattus*) and 8.3% in livestock (13.5% in cattle and 12.6% in goats). The prevalence among rodents and livestock differed between settlements (*p* = 0.01). A higher prevalence was observed among rodents in the agricultural settlement relative to the other settlements. A higher prevalence of antibodies in livestock was observed in the livestock settlement compared with the mixed agricultural–livestock settlement. The *Leptospira* serovars Sokoine (serogroup Icterohaemorrhagiae) and Hebdomadis (serogroup Hebdomadis) were detected in both rodents and livestock. The serovars Hardjo (serogroup Sejroe) and Gripothyphosa (serogroup Gripothyphosa) were found exclusively in cattle, whereas the serovars Pomona (serogroup Pomona) and Lora (serogroup Australis) were identified in rodents. *Leptospira* antibodies were found to be elevated during the rainy season compared with the dry season (*p* = 0.05) in all settlements, with the exception of rodents in the mixed agricultural–livestock settlement. Conclusions: This study demonstrates the presence of anti-*Leptospira* antibodies in rodents and livestock related to occupational activities in human settlements. It further demonstrates that wild animals (rodents) and livestock are reservoirs of *Leptospira* and are important in the epidemiology of leptospirosis. Management and control strategies should target both rodents and livestock.

## 1. Introduction

Leptospirosis is an important zoonotic disease that can be transmitted from animals to humans [1]. The disease is of public health concern, especially in tropical and subtropical areas, where favorable climatic and environmental factors can facilitate the persistence and transmission of the bacteria [2]. The disease impacts both humans and animals, presenting a wide range of clinical symptoms in humans, ranging from mild, flu-like symptoms to severe conditions, such as jaundice, renal failure, and pulmonary hemorrhage, which may be fatal if left untreated [3]. Leptospirosis in livestock can result in reproductive failure, reduced milk production, and mortality, contributing to significant economic losses [4]. A good understanding of the epidemiology of leptospirosis in endemic regions is essential in order to reduce human and animal infections [5].

Wetlands are recognized as high-risk zones for leptospirosis transmission, attributed to their warm and humid conditions that promote the survival and proliferation of *Leptospira* [6,7]. This kind of environment also favors numerous mammalian species, particularly rodents. Rodents are particularly important in the epidemiology of Leptospirosis since they are the primary reservoirs of the bacteria, facilitating the maintenance and dissemination of the infection both within and among species [8]

The wetlands of Kilombero, Tanzania, are characterized by intensive agricultural activities, limited pastoralism, and various human occupations, creating a distinct ecological niche that facilitates frequent interactions between humans and animals [9]. This environment increases the potential for zoonotic disease transmission, in particular, leptospirosis from the abiotic environment and animals, such as rodents, to humans [10]. In the Kilombero wetlands, various occupational activities, such as irrigated rice farming, fishing, and livestock rearing, expose individuals to water and soil that may be contaminated with *Leptospira* [11], which can increase the risks of transmission of *Leptospira* within the ecosystem. Studies on the prevalence of *Leptospira* in animal reservoirs in Kilombero remain limited. A few studies in the past were focused on its prevalence among febrile patients [12]. There is, therefore, a lack of a clear understanding of the epidemiology of leptospirosis, which is critical knowledge necessary for developing targeted interventions and control strategies for the disease in Tanzania.

To address this gap, it is essential to assess the seroprevalence of *Leptospira* spp. in both livestock and rodents in Tanzania’s wetlands, considering the various occupational activities that may influence the risks of exposure. This study investigated the seroprevalence of *Leptospira* spp. associated with rodents as primary reservoir hosts, livestock, and human settlements (occupational activities) in the wetlands of the Kilombero Valley in the Morogoro region, Tanzania.

## 2. Materials and Methods

### 2.1. Area of Study

**Figure 1:**
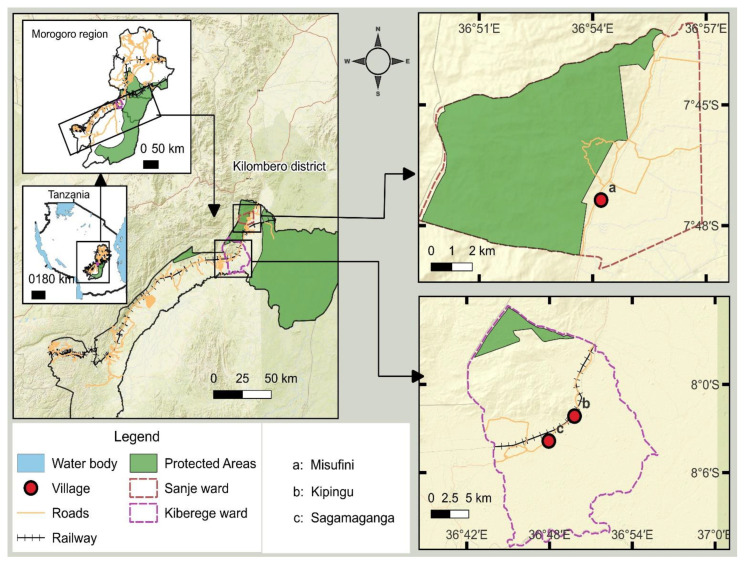
Map illustrating the geographical position of the study villages within the Kilombero wetland, in the Kilombero district, Tanzania. a = Misufini Village (agriculture-based settlement); b = Kipingu Village (livestock-based settlement), and c = Sagamaganga Village (mixed agricultural–livestock settlement).

This study was conducted in the wetland area of the Kilombero Valley, situated in the Kilombero district in the Morogoro region, Tanzania (Figure 1). We selected human settlements based on their main occupational activities, which were considered to possibly influence the prevalence of *Leptospira*. The first settlement, Misufini Village, is primarily agricultural, with both rainfed and irrigated rice, with rice fields close to homesteads and no livestock. This settlement was classified as agriculture-based. The second settlement, Kipingu Village, is predominantly inhabited by livestock keepers, the main livestock being cattle and goats. Some residents also engage in the cultivation of rice and maize, mainly rainfed and, therefore, seasonal, with the farms being far from the homesteads. The settlement was classified as livestock-based. The third settlement, Sagamaganga Village, is a mixed agricultural and livestock keeping. The crops are rainfed, with farms close to homesteads, and the livestock are mainly goats. The settlement was classified as mixed farming and livestock.

### 2.2. Rodent Trapping and Sample Collection

Samples were collected once in the rainy season (February 2023) and once in the dry season (August 2023). Rodents were sampled in both settlements; however, livestock sampling occurred exclusively in the mixed and livestock-based settlements. Rodents were captured in houses, peridomestic surroundings, and agricultural fields using Sherman live-capture traps (Sherman^®^ traps: 7.5 × 9.0 × 23.0 cm; HB Traps, Inc., Tallahassee, FL, USA) and locally made wire cage traps. The distribution of the traps was as follows: one Sherman and one wire cage trap were placed inside houses and in peridomestic areas, while four Sherman and four wire cage traps were set in agricultural fields. The traps were baited with a mixture of maize flour and peanut butter and were left at the trapping station for four consecutive nights. Every morning, the traps were inspected, and captured animals were removed and placed individually in a cloth bag and labeled for further processing in a field laboratory. Data on the capture locality were recorded.

In the field laboratory, the following data were recorded: species identity, morphological data (body weight, head and body length, and tail length), sex and reproductive condition (male: scrotal or abdominal testes; female: a perforated or closed vagina, and pregnant or lactating) were recorded. Blood samples (0.5–1 mL) were obtained through cardiac puncture and were centrifuged at 4000 rpm for 5 min to isolate sera and subsequently stored at −20 °C for further analysis [13].

### 2.3. Livestock Sample Collection

Blood samples were collected from the jugular vein of each animal using a sterilized syringe and needle. An amount of 10 mL of blood was obtained per animal and centrifuged in the field, and the resulting sera were stored at −20 °C for subsequent analysis. Samples were collected from 20 households in the livestock-based settlement. For each household, two cattle and two goats were randomly selected for blood collection (a total of 80 blood samples). In the mixed agricultural–livestock settlement, five households were randomly selected, and from each of them, blood samples were taken from four goats (a total of 20 blood samples). Sampling was performed once in the rainy season (February 2023) and once in the dry season (August 2023). A total of 200 livestock blood samples (cattle and goats) were collected from the livestock and mixed farm–livestock settlements.

### 2.4. Detection of Anti-Leptospira Antibodies Using Microscopic Agglutination Test (MAT)

We used the microscopic agglutination test (MAT), a gold standard for *Leptospira* serology and classification [14]. A panel of known *Leptospira* serovars of both local isolates and foreign origins as reference was used. For this study, live cultures from the *Leptospira* serogroup Hebdomadis (*Leptospira santarosai* serovar Hebdomadis), Icterohaemorrhagie (*Leptospira interrogans* serovar Sokoine), Sejroe (*Leptospira interrogans* serovar Hardjo), Grippotyphosa (*Leptospira kirschneri* serovar Grippotyphosa), Pomona (*Leptospira interrogans* serovar Pomona), Australis (*Leptospira interrogans* serovar Lora), and Canicola (*Leptospira interrogans* serovar Canicola), were used. These serovars are known to infect both rodents and livestock and are prevalent in Tanzania [15,16,17]. The serovars were inoculated into Ellinghausen and McCullough medium modified by Johnson and Harris (EMJH) and incubated at 30 °C for 5–7 days. Fully grown *Leptospira* cultures, with a density of 300 × 10^8^ leptospires/mL, as determined by McFarksley, were used as reference antigens in the MAT.

For the screening, 10 μL of each sample was serially diluted in 90 μL of phosphate-buffered saline (pH 7.0) at dilutions of 1:10, 1:20, and 1:80. Afterward, 50 μL of live leptospire antigen was added to all wells containing serially diluted serum, thus increasing the dilutions to 1:20, 1:40, and 1:160. Samples that tested positive during screening were subjected to titration at various dilutions (i.e., 1:10, 1:20, 1:40, 1:80, 1:160, 1:320, 1:640, 1:1280, etc.) [18]. The positive samples were identified by observing the cut-off point of >1:160, at which 50% of *Leptospira* exhibited agglutination [8,19,20]. This was conducted using a dark-field microscope. Each test incorporated negative and positive controls. Phosphate-buffered saline (PBS) was used as a negative control, whereas positive controls were assigned to each unique testing serovar.

### 2.5. Analysis

The prevalence of anti-*Leptospira* antibodies in rodents and livestock was determined as the ratio of infected animals to the total number collected, categorized by the species of the rodents and livestock as well as the settlement/village categories. The locality-specific prevalences of *Leptospira* associated with rodents and livestock were assessed using the Fisher exact test. The seroprevalence of *Leptospira* serovars in rodents and livestock was calculated as the percentage of infected animals with specific serovars against the number of animals sampled. The variations across serovars in different settlements/villages were assessed using the Fisher exact test, while the seasonal effects on cattle and rodents across settlements/villages were also evaluated using the Fisher exact test. All statistical analyses were conducted using the R software, version 4.3.3.

## 3. Results

### 3.1. Rodent and Livestock Population Analysis/Demography

A total of 179 rodents were live captured in the three settlements. Of these, 46% (n = 84/179) were collected from the agriculture-based settlement, with 32% (n = 59/179) captured in February 2022 and 13% (n = 25/179) in August 2023. In the livestock settlement, 29.6% (n = 53/179) of rodents were captured, with 29% (n = 52/179) collected in February 2023 and 1 in August 2023. In the mixed agricultural–livestock settlement, 13% (n = 42/179) of rodents were captured, with 20% (n = 36/179) collected in February 2023 and 0.3% (n = 6/179) in August 2023.

The rodent captures comprised two species, of which 51% (n = 92/179) were *multimammate rats *(*Mastomys natalensis*) and 48% (n = 87/179) were black rats (*Rattus rattus*). The distribution of the rodent captures within settlements was 30% (n = 54/179) in agricultural fields, 52% (n = 94/179) in households, and 17% (n = 31/179) in the peridomestic environment. Most of the rodents captured were adults, at 78% (n = 141); the rest comprised juveniles, at 21% (n = 38). The percentages of female and male captures were 53.1% (n = 95/179) and 46.9% (n = 84/179), respectively.

For the livestock, a total of 200 animals were sampled, consisting of 40.0% (n = 80/200) cattle and 60.0% (n = 120/200) goats. In the livestock settlement/village, a total of 160 animals were sampled, consisting of 80 cattle and 80 goats. A total of 40 goats were sampled in the mixed agricultural–livestock settlement/village; cattle were not reared in this settlement.

### 3.2. Prevalence of Leptospira Antibodies in Rodents and Livestock Across Settlements/Villages

**Figure 2:**
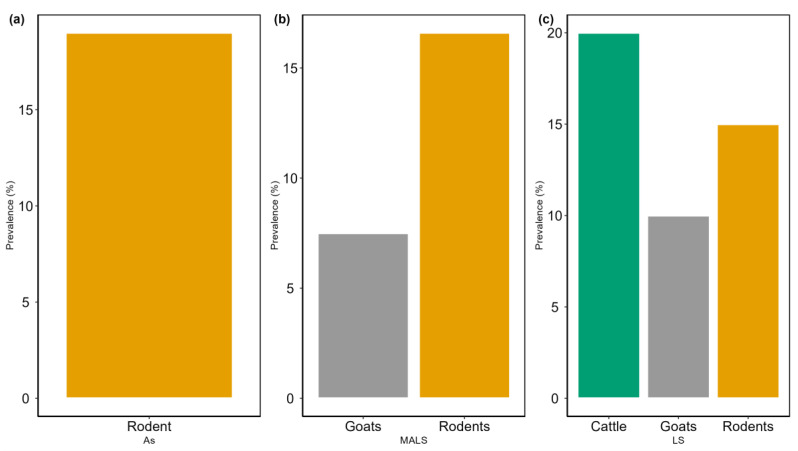
Variations in the prevalence of *Leptospira* seropositivity in rodents and livestock in three different settlements in the Kilombero Valley, Tanzania. (As: agricultural settlement, MALS: mixed agricultural–livestock settlement, and LS: livestock settlement). (**a**) Rodent prevalence, (**b**) Goats and Rodent prevalence (**c**) Cattle, Goats and Rodent prevalence.

The prevalence of *Leptospira* antibodies differed between rodent species and livestock, with the highest (17.3%) in rodents compared with livestock (13.5%). Within the livestock settlement, the prevalence of seropositive *Leptospira* was significantly higher (*p* = 0.001) in livestock (20.0% and 10.0% for cattle and goats, respectively) than in rodents (15.0%) (Figure 2). In the mixed agricultural–livestock settlement, the prevalence was significantly higher (*p* = 0.001) in rodents (16.6%) than in goats (7.5%). In the agricultural settlement, the prevalence in rodents was 19% (n = 16/84) (Figure 2).

The prevalence of *Leptospira* antibodies was highest in *M. natalensis* (21.7%; n = 20/92) relative to *R*. *rattus* (12.6%; n = 11/87). There were variations in *Leptospira* antibodies’ prevalence in rodents associated with the settlement type. The prevalence in rodents was significantly higher (*p* = 0.001) in the agricultural settlement than in the mixed agricultural–livestock and livestock settlements. There were no significant differences observed in *Leptospira* seropositivity between crop vegetation areas (25.9%; n = 14/54) and peridomestic areas (28.1%; n = 9/32). However, a significantly lower (*p* = 0.001) prevalence was observed in houses (8.6%; n = 8/92) (Table 1).

### 3.3. Seroprevalence of Leptospira Serovars

The seropositivity of rodents for the *Leptospira* serovars Sokoine (serogroup Icterohaemorrhagiae) and Hebdomadis (serogroup Hebdomadis) was 8.3% (n = 15/179) and 3.9% (n = 7/179), respectively, whereas the same serovars had a seropositivity of 4.5% (n = 9/200) and 3% (n = 6/200) in livestock. The serovars Hardjo (serogroup Sejroe) and Gripothyphosa (serogroup Gripothyphosa) were exclusively found in cattle with a seropositivity of 5.5% (n = 11/200) and 1.5% (n = 3/200), respectively. The serovars Pomona (serogroup Pomona) and Lora (serogroup Australis) were detected in rodents only with a seropositivity of 1.6% (n = 3/179) and 3.3% (n = 6/179), respectively. The serovar Canicola was not detected in either rodents or livestock (Table 2).

There were some variations in seropositivity associated with the type of settlement. Rodents in the agricultural settlement had a higher prevalence of the serovars Sokoine (8.3%; n = 7/84) and Lora (7.1%; n = 6/84) and a low prevalence of serovar Hebdomadis (2.3%; n = 2/84). In the mixed agricultural–livestock settlement, rodents were infected with the serovars Sokoine (11.9%; n = 5/42) and Pomona (7.1%; n = 3/42). In the livestock settlement, rodents were more infected with the serovars Hebdomadis (9.4%; n = 5/53) and Sokoine (5.6%; n = 3/53).

**Figure 3:**
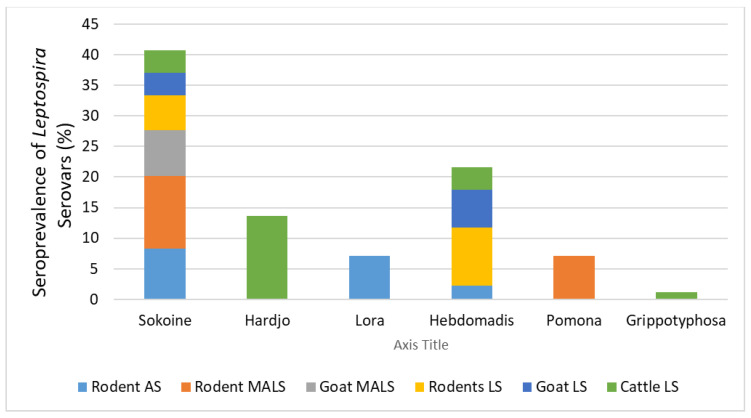
Seropositivity in relation to *Leptospira* serovars detected in rodents, cattle, and goats from three settlements/villages. As: agricultural settlement, MALS: mixed agricultural–livestock settlement, and LS: livestock settlement.

The seroprevalence of *Leptospira* spp. found in goats in the mixed agricultural–livestock settlement was the serovar Sokoine (7.5%; n = 3/40), whereas goats were positive for the serovars Sokoine (3.7%; n = 3/80) and Hebdomadis (6.2%; n = 5/80) in the livestock settlement. Cattle in the livestock settlement were predominantly infected with the serovars Hardjo (13.7%; n = 11/80), Hebdomadis (3.7%; n = 3/80), Gppotyphosa (1.25%; n =1/80), and Sokoine (3.7%; n = 3/80) (Figure 3).

### 3.4. Seasonal Prevalence of Leptospira Antibodies

**Figure 4:**
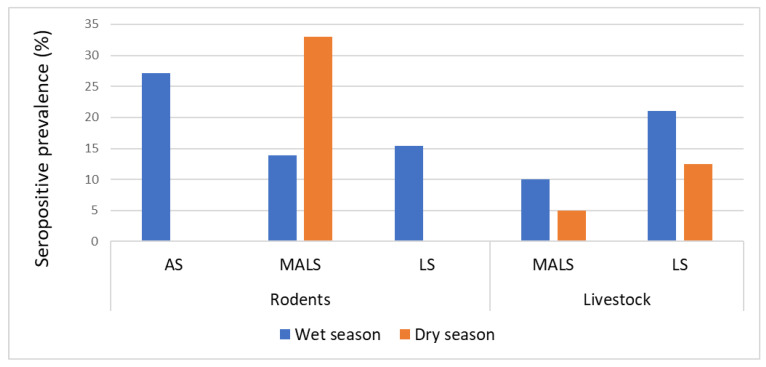
Seasonal differences in the prevalence of anti-*Leptospira* antibodies in agricultural, mixed agricultural–livestock, and livestock settlements in the Kilombero Valley, Tanzania. (As: agricultural settlement, MALS: mixed agricultural–livestock settlement, and LS: livestock settlement).

There were significant variations in the seropositivity of *Leptospira* in livestock (*p* < 0.5) and rodents (*p* < 0.05) in the three settlements associated with seasonality. In the livestock settlement, the prevalence was higher during the rainy season (18.7%; n = 15/80) relative to the dry season (12.5%; n = 10/80). In the mixed agricultural–livestock settlement, the prevalence in goats was 10% (n = 2/20) and 5% (n = 1/20) in the rainy and dry seasons, respectively (Figure 4).

For rodent species, more positive cases were identified during the rainy season in both agricultural and livestock settlements. In the mixed agricultural–livestock settlement, the prevalence of *Leptospira* antibodies in rodents was higher during the dry season compared with the rainy season (Figure 4).

## 4. Discussion

This study investigated the seroprevalence of *Leptospira* spp. in rodents and livestock (cattle and goats) across three settlements/villages characterized by the dominance of three different occupational activities, namely, agriculture, livestock keeping, and a combination of both.

In the livestock-based settlements where both rodents and livestock were sampled, the prevalence of seropositive *Leptospira* was higher in cattle than in rodents, followed by goats. This suggests livestock are important sources of infection of *Leptospira* in communities where livestock keeping is an occupational activity [21,22]. Primary reservoirs of *Leptospira* could potentially infect and discharge the bacteria into the environment from which the cattle and goats get infected when feeding. Humans probably get infected when they consume undercooked meat or milk that has not been boiled [23,24]. The higher seroprevalence of *Leptospira* in cattle than in goats could be attributed to feeding habits. Cattle are grazers, and, therefore, they are more likely to become infected by *Leptospira* from the soil [25].

The lower seroprevalence of *Leptospira* in rodents in livestock settlements can be attributed to the wider foraging range of livestock [26] and the increased risk of getting infected at water ponds. Rodents, in contrast, have a restricted home range determined by their habitat preferences [27].

The higher seroprevalence of *Leptospira* in goats in livestock settlements than in mixed agricultural–livestock settlements is probably due to different livestock management practices. In livestock settlements, goats are free-roaming and have frequent contact with other infected animals and contaminated environments, hence elevating their infection risk [28]. The increased prevalence of rodents in agricultural settlements relative to mixed agricultural–livestock and livestock settlements suggests that rodent abundance significantly influences infection dynamics [29,30]. A higher abundance of rodents in an agricultural settlement certainly increases the infection rate among rodents. An increase in the number of rodents most likely elevates the contamination of the environment and transmission between individuals [31,32].

Among rodent species, * M. natalensis* had a higher seroprevalence rate than *R. rattus*, indicating that *M. natalensis* may have a greater and more substantial role in the maintenance and dissemination of *Leptospira* serovars in the three types of settlements. *M. natalensis* has higher mobility than *R. rattus* and frequently burrows in the ground, potentially exposing it to contaminated urine or waste from soil and water [33]. *R. rattus* is usually more active on roofs [34], which, therefore, renders it less vulnerable to encounters with *Leptospira* on the ground. *M. natalensis* was the most common in all three settlements but was more abundant in the agricultural settlement within crop fields and peridomestic areas where there was a higher seroprevalence of *Leptospira* than in houses. The lower prevalence of *Leptospira* in houses could be due to an environment that was less favorable to infestation by rodents and the maintenance of good hygienic conditions, which do not favor contamination with *Leptospira* and transmission [35]. The maintenance of dry conditions in houses does not favor the survival of *Leptospira* and reduces the risk of human infections [35,36].

The serovars Sokoine and Hebdomadis were the most prevalent in both rodents and livestock, but they have also been documented in multiple locations and isolated from diverse animals in Tanzania, including freshwater fish [37], domestic animals, such as cattle, goats, and sheep [38,39], wild animals, [20], and humans [40]. The serovar Sokoine was found in all three settlements in rodents and livestock. The serovar Sokoine has been isolated in various regions of Tanzania [41], across multiple animal species [37,42,43], and has been reported in Kenya [44].

The serovars Hardjo and Gripotyphosa were found in cattle and showed host specificity. The serovar Hardjo is recognized as the primary *Leptospira* responsible for significantly impacting cattle output and serving as the principal cause of infection in humans. This serovar has been recorded in many regions of Tanzania, including the northern regions [5], the southern highlands [45], and the western regions [20]. The absence of Grippotyphosa in rodents and Lora and Pomona in livestock may be attributed to the prevailing environmental and climatic conditions during the time of our study; these serovars have been documented in previous studies involving rodents and livestock [8].

The serovar Sokoine was the most dominant in rodents, with the exception of the livestock settlement, whereas Hebdomadis was more prevalent in livestock and rodents in this settlement. This distribution pattern is probably determined by the abundance of hosts, in this case, the higher abundance of cattle. The predominance of the serovar Hebdomadis in livestock, particularly cattle, suggests that environmental and animal husbandry practices in livestock-based villages may facilitate the transmission of this serovar [5]. The coexistence of multiple serovars in animals within a settlement can be attributed to host specificity and/or infection of the same animal species by different serovars. For instance, the serovar Hardjo is unique to cattle, but other serovars may inadvertently infect cattle due to their presence in an area or being shed by other species, such as rodents [45].

The survival of *Leptospira* is highly influenced by climatic factors and is more prevalent in areas characterized by moderate temperatures, substantial rainfall, and elevated humidity [46]. Our study demonstrated an increased seropositivity during the rainy (wet) season relative to the dry season in both rodents and animals from agricultural and livestock settlements. Precipitation facilitates the transfer of leptospires from contaminated soil, feces, or urine into water sources accessible to livestock during the rainy season [47]. In rodents, the rainy season leads to increased water availability in agricultural fields, resulting in a moist environment that enhances *Leptospira* survival, while increased resources for rodents enhance reproduction, population abundance, and an increase in bacterial circulation.

## 5. Conclusions

This research highlights the prevalence of *Leptospira* antibodies in rodents and livestock in three settlements/villages characterized by different occupational activities. The high prevalence of *Leptospira* antibodies in rodents in agricultural settlements suggests that targeted interventions, including improved water management strategies and enhanced rodent control measures, are necessary. Mixed agricultural livestock settlements may benefit from initiatives aimed at addressing both agricultural and residential infection sources. In livestock-based settlements, while rodents are less abundant, the high prevalence of infection in cattle must be taken into account, as cattle may be the main source of infection in humans. The significant differences in infection rates across various species of animals and village types highlight the need for appropriate strategies for the prevention of *Leptospira* infection, which must consider local epidemiological conditions, host species, whether wild or livestock, and occupational activities that render or increase the risk of human infections.

## Figures and Tables

**Table 1:** The prevalence of *Leptospira* seropositivity in rodents (*M. natalensis* and *R. rattus*) captured in houses, peridomestic surroundings, and agricultural fields in three settlements/villages in the Kilombero Valley, Tanzania.

	Agricultural		Settlement/Village Category Mixed (Agricultural–Livestock)		Livestock	
	* M. natalensis *	* R. rattus *	* M. natalensis *	* R. rattus *	* M. natalensis *	* R. rattus *
Agricultural fields	18.4% (7/38)	0 (0/0)	25% (3/12)	100% (1/1)	100% (3/3)	0% (0/0)
Peridomestic surroundings	11.5% (3/26)	50% (1/2)	100% (2/2)	100% (1/1)	0% (0/0)	100% (1/1)
In houses	20% (2/10)	37.5% (3/8)	0% (0/1)	0% (0/25)	0% (0/0)	8.1% (4/49)

**Table 2:** *Leptospira* serovars and their corresponding serogroups identified from livestock and rodents in the Kilombero Valley, Tanzania.

		Livestock		Rodents	
		Cattle	Goats	* M. natalensis *	* R. rattus *
*Leptospira* Serovar	Serogroup				
Sokoine	Icterohaemorrhagiae	3.7% (n = 3/80)	5% (n = 6/120)	7.6% (n = 7/92)	9% (n = 8/87)
Hebdomadis	Hebdomadis	3.7% (n = 3/80)	4% (n = 5/120)	4.3% (n = 4/92)	3.4% (n=3/87)
Lora	Australis	0	0	6.5% (n = 6/92)	0
Gripothyphosa	Gripothyphosa	1.2% (n = 1/80)	0	0	0
Canicola	Canicola	0	0	0	0
Pomona	Pomona	0		3.2% (n = 3/92)	0
Hardjo	Sejroe	13% (n = 11/80)	0	0	0

## Data Availability

The data supporting the reported results can be made available from the Institute of Pest Management (IPM) upon request.
